# Embedding and Publishing Interactive, 3-Dimensional, Scientific Figures in Portable Document Format (PDF) Files

**DOI:** 10.1371/journal.pone.0069446

**Published:** 2013-09-25

**Authors:** David G. Barnes, Michail Vidiassov, Bernhard Ruthensteiner, Christopher J. Fluke, Michelle R. Quayle, Colin R. McHenry

**Affiliations:** 1 Monash Biomedical Imaging, Monash University, Clayton, Victoria, Australia; 2 Monash e-Research Centre, Monash University, Clayton, Victoria, Australia; 3 Faculty of Information Technology, Monash University, Clayton, Victoria, Australia; 4 Institute of Asian and African Studies, Moscow State University, Moscow, Russia; 5 Section Evertebrata varia, Zoologische Staatssammlung München, München, Bavaria, Germany; 6 Centre for Astrophysics and Supercomputing, Swinburne University of Technology, Hawthorn, Victoria, Australia; 7 Department of Anatomy and Developmental Biology, School of Biomedical Sciences, Monash University, Clayton, Victoria, Australia; Banner Alzheimer's Institute, United States of America

## Abstract

With the latest release of the S2PLOT graphics library, embedding interactive, 3-dimensional (3-d) scientific figures in Adobe Portable Document Format (PDF) files is simple, and can be accomplished without commercial software. In this paper, we motivate the need for embedding 3-d figures in scholarly articles. We explain how 3-d figures can be created using the S2PLOT graphics library, exported to Product Representation Compact (PRC) format, and included as fully interactive, 3-d figures in PDF files using the movie15 LaTeX package. We present new examples of 3-d PDF figures, explain how they have been made, validate them, and comment on their advantages over traditional, static 2-dimensional (2-d) figures. With the judicious use of 3-d rather than 2-d figures, scientists can now publish, share and archive more useful, flexible and faithful representations of their study outcomes.

*The article you are reading does not have embedded 3-d figures. The full paper, with embedded 3-d figures, is recommended and is available as a supplementary download from PLoS ONE (File S2).*

## Introduction

### The visualisation of scientific data is important

Visualisation is used throughout the scientific workflow: in experiment design, data acquisition, data comprehension and analysis, and importantly, in the communication of outcomes. In the era when scientific data is increasingly multi-dimensional in nature, 3-dimensional (3-d) data rendering and display tools are mainstream, and indeed in some application areas (e.g. geology) are now considered essential. In addition to the simple 3-d extensions of standard 2-dimensional (2-d) plots (e.g. scatter plots, histograms, bar graphs), specific 3-d renderings such as isosurfaces (an analog of 2-d contours) [1], streamlines (an analog of 2-d vector/flow plots), and volume rendering [2] exist. However, due to the static, 2-d nature of the two major contemporary publishing media (paper and screen), the communication and publication of research outcomes still depends routinely on the standard 2-d plots, and less frequently, 2-d projections of 3-d renderings.

### Static projections of 3-d data to a flat medium are useful though

The human visual system can convincingly and accurately interpret appropriately-drawn static figures on 2-d paper or screen as 3-dimensional. Notwithstanding the classic Necker wireframe cube illusion [3], the cognitive system can make sophisticated assumptions about 3-d scenes projected as flat 2-d illustrations. One-point perspective rendering can provide a very strong depth cue (although distorting object proportions), while providing more than one projection of the same 3-d figure (onto non-parallel projection planes) gives the observer the opportunity to manually assemble or improve the model of the scene in their mind. For most people, simple stereoscopic rendering approaches such as using a red-cyan anaglyph can further improve 3-d perception, to the extent that highly-accurate depth-ordering can be interpreted by the brain, and even quantitative estimates of depth can be made with reasonable certainty. However, anaglyphs and other stereoscopic techniques such as ChromaDepth (American Paper Optics, Inc.—http://www.chromatek.com/) are rarely used in formal publishing due to the need for viewing accessories (e.g. red-cyan glasses, stereoscope) and their restrictive impact on the use of colour.

### Dynamic projections of 3-d data to the screen are vastly more useful

Most contemporary visualisation software provides three crucial features for examining 3-d renderings:

1. motion parallax (via auto-rotation of the camera or small-angle changes in the viewing position);
2. interactive, unconstrained camera control; and
3. toggling of components of the visualisation.

Motion parallax is a dominant depth cue (e.g. [4] and references therein), and together with interactive control of the camera position and viewing direction, can substantially enhance the natural comprehension of spatial relationships in the data. Freestyle camera control can also enable the observation of features that might otherwise be concealed in certain fixed views of the data, and switching components in and out of the visualisation can reveal features that are concealed *within* other features (e.g. high value isosurfaces within opaque, low value isosurfaces). None of these *interactive* (i.e. viewer-controlled) features can be replicated in static (still) images on paper or the screen.

### Researchers can already share dynamic 3-d visualisations, albeit on an ad hoc basis

However, there is a substantial impediment to doing this, and it remains difficult to accomplish within the confines of the present-day professional communication and publication process. The data owner must: (a) publish their data (either raw or processed) usually in a disconnected fashion with respect to the destination of their formal research article; (b) recommend and/or make available computer programs (preferably Free Software; in this paper, we use the term *Free Software* as defined by the Free Software Foundation: http://www.fsf.org/) for reading and displaying the data; and (c) explicitly document the steps to producing the exact visualisation shown in the paper, possibly from a large pool of raw data. The community member who wishes to reconstruct the dynamic visualisation needs to (d) retrieve a potentially large data set; (e) install all requisite software on a compatible system; and (f) follow potentially myriad lengthy steps to reproduce the graphic. An explicit example of the effort involved in sharing visualisations of 3-d protein structures is given by [5]. Certainly some community members will already have the requisite software, but others working in the same discipline but with different software tools may not, and others beyond the particular discipline may simply be unable to reproduce the dynamic visualisation due to lack of domain-specific knowledge, appropriate software and experience.

### Standardised, 3-d geometry languages can be used to improve sharing

An obvious alternative to the above is to publish an intermediate dataset that is effectively “geometerised” data. A transfer function from data space to 3-dimensional space is applied to the data, yielding a set of geometry that may comprise points (vertices), lines and facets that connect vertices, and surfaces made up of multiple interconnected facets. The geometry is stored in a standard format file and can be displayed by one or more software tools. Many standardised languages exist, and numerous authors (including one of us) have deployed 3-d data in this manner (e.g. [6], [7]). Yet even the most well-known of these languages—Virtual Reality Markup Language (VRML97)—remains a somewhat unreliable solution, with dozens of incomplete implementations in mainstream use all but guaranteeing differing results on different viewing systems. Even if identical, complete implementations of a viewer existed for every operating system in common use, the fact remains that publishing 3-d data in this way leaves it significantly disconnected from the literature, relegated to surviving as “supplementary material”, a second-class illustration not strictly necessary for complete comprehension of the article.

A few authors have recognised the need to publish a single file containing text, tables, graphs *and* 3-d interactive figures. Notably, the *iSee* approach [8] unifies descriptive text, static images, and interactive 3-d visualisation(s) [5] in a single file. Although driven by the needs of the structural biology community and the authors' stated desire to enable the “communication of complex structural biology and related data to a wide audience of non-structural biologists”, the iSee strategy is clearly translatable to other disciplines such as the physical sciences. However, as a solution built on a custom, specialised, non-archival data format and a custom browser, the authors have perpetuated the need for new users to download, install and learn how to use non-standard software. They have also implicitly taken on the task of maintaining a cross-platform document browser that works in and out of the traditional web-browser environment.

### Three-dimensional data should be published in interactive, 3-d form, without requiring *any* special viewing software or even plugin: specifically, 3-d figures should be published *in situ*. Custom data formats and software plug-ins for the reader should be eschewed

The CAD industry has much the same needs. To serve them, Adobe Systems Inc (hereafter, Adobe) extended its well-established Portable Document Format (PDF) with built-in support for standard-based 3-d models (as opposed to third-party plug-ins, visualizing data in proprietary formats), so as to offer a universal solution for publishing engineering documentation. In Acrobat 3D, Adobe provided a relatively simple environment for importing 3-d geometry from a great number of standard 3-d formats, for configuring lighting and shading properties, and for predefining specific views. Acrobat Reader — the standard application for reading PDF documents on the major contemporary computer platforms (the Microsoft Windows family, Linux and Mac OS X) — could immediately display the 3-d rendering to the user and provided free-style interactive camera control. The model tree could be navigated and different components of the 3-d scene toggled in and out of the display.

Even though Adobe's work appeared to be principally motivated to support the sharing of 3-d engineering and design drawings, several authors (including us) have used the technology to embed and publish (in the scientific literature) interactive, 3-d figures of scientific data in PDF files. Like others, our first successes [9]–[11] made use of (commercial) Adobe software (Adobe Acrobat 3D version 8) to read VRML97 scenes, in our case written by our own software library S2PLOT [12]. Other authors (e.g. [13]) used 3rd-party commercial software such as Right Hemisphere's “Deep Exploration” visualisation package. (Adobe Acrobat 3D has been superceded by Adobe Acrobat X Pro, and in-built support for adding 3-d figures was dropped — see File S1. A third-party plug-in from Tetra 4D is required to create 3-d PDF figures using the approach previously described ([9]–[11]).) The created 3-d figures can then be embedded in existing PDF documents made by other applications. In the context of writing a scientific article, the result can be a single, compact file comprising text, graphs, figures, tables and 3-d interactive figures, readable by the *de facto* standard PDF reader application — Adobe Reader.

### In this paper we announce the release of our S2PLOT graphics library as Free Software, and describe the open-source pathway that this enables for embedding 3-d figures in PDF documents

To improve uptake by the scholarly community, we have developed a process for generating 3-d PDF figures using Free Software, integrated directly with the widely-used LaTeX typesetting system. Our complete workflow is not implemented as a pretty graphical “swiss army knife” visualisation program, rather it is an addition to the S2PLOT library that enables, for program codes using the S2PLOT library for visualisation: (i) output of S2PLOT visualisations as PRC-format files for inclusion in LaTeX -generated PDF files, and (ii) direct output of PDF files. It is more flexible than the commercial path, includes support for common scientific visualisation techniques (e.g. volume rendering) that are difficult to produce using the commercial tools, and integrates well into the scientific publishing workflow. PDF files created with our method can be viewed with any PDF viewer, but embedded 3-d figures are shown only when viewed with Adobe Reader or Adobe Acrobat on a desktop platform.

## Methods

### Tools and technologies

#### The Adobe Product Representation Compact (PRC) format

The Adobe PRC format (http://livedocs.adobe.com/acrobat_sdk/9/Acrobat9_HTMLHelp/API_References/PRCReference/PRC_Format_Specification/) stores hierarchically-structured 3-d data, whose constituent parts are 3-d vector entities such as vertices, lines and facets. Extensive description of part colours, material properties and texture maps is possible. PRC is one of only two native 3-d scene description file formats that can be embedded in PDF files and subsequently displayed by Adobe Reader. While the PRC format is documented, and an ISO process is being followed for standardization of PRC (ISO standardization: ISO/DIS 14739-1 “Document management – 3D use of Product Representation Compact (PRC) format – Part 1: PRC 10001”; ISO/DIS 14739-1.2: http://www.iso.org/iso/iso_catalogue/catalogue_tc/catalogue_detail.htm?csnumber=54948), no reference implementations of a library to read and write PRC files exists to our knowledge. See File S1 for a concise history of the Adobe-3d PDF and the two formats PRC and U3D.

#### Adobe 3D JavaScript

Adobe Reader includes a JavaScript interpreter that is traditionally used to support PDF forms and user-interaction in PDF documents. For 3-d figures, the JavaScript interpreter has been enhanced to provide access to the scene model tree, the scene rendering settings, and to respond to user actions on the 3-d figure. For example, changes to the camera position can be captured and acted upon. JavaScript for Acrobat 3D is well-documented (http://www.adobe.com/devnet/acrobat/pdfs/js_3d_api_reference.pdf).

#### Asymptote: the Vector Graphics Language

Asymptote, the Vector Graphics Language (http://asymptote.sourceforge.net/), is a sophisticated vector language for describing 2-d and 3-d graphics and technical drawings. Asymptote can generate PRC files containing 3-d scenes, and embed them within PDF files by making external calls to LaTeX. A number of elegant examples are provided that show Asymptote's ability to render 3-d surfaces and graphs in PDF documents (http://asymptote.sourceforge.net/gallery/3D graphs/). Asymptote is licensed under the GNU Lesser Public License, and is our source for a freely-available PRC writing library. One of us (MV) developed the PRC export code in Asymptote.

#### The movie15 LaTeX package

The movie15.sty style file (http://www.ctan.org/tex-archive/macros/latex/contrib/movie15/; recently superceded by media9.sty (http://www.ctan.org/tex-archive/macros/latex/contrib/media9)) extends LaTeX with the capability to insert various non-static media in PDF files created with LaTeX. Specifically it can embed PRC-format files as 3-d figures, with optional 3D JavaScript attachments that are enabled on activation of the 3-d figure. It also provides mechanisms to create preset views, shading and lighting options for 3-d figures.

#### The libHaru free PDF library

LibHaru (http://libharu.org) is a freely-available library that provides an application programing interface to generate PDF files, comprising text, vector graphics and images, and 3-d figures based on PRC-format (and U3D-format) files and optional 3D JavaScript scripts. Like the movie15 LaTeX package, libHaru provides hooks to create preset views, shading and lighting options for 3-d figures.

#### The S2PLOT 3-d graphics library

The S2PLOT library (http://astronomy.swin.edu.au/s2plot) [12], which we announce in this paper to be available as Free Software, provides a clean and simple API for creating interactive, 2-d and 3-d visualisations of data. S2PLOT's rich API of more than 200 functions includes support for low-level geometric primitives (points, lines, 3- and 4-vertex facets), intermediate-level structures (disks, cylinders, cones, ellipsoids, vector and bitmap text, textured facets including billboards), and high-level science-oriented entities such as axis labels, surface and skyscraper plots, isosurfaces and volumetric renderings. Camera interaction and control, and rendering settings are standard to all S2PLOT programs, but can be modified by the programmer. A simple but flexible callback system supports the display of time-dependent or animated geometry and the receipt of user input including 3-d object selection and manipulation.

From version 3.1 upwards, S2PLOT can write PRC files using the included PRC export module, based on the PRC export code in Asymptote. Nearly all types of geometry rendered interactively by S2PLOT can be exported to a PRC file. Noteworthy features of the S2PLOT PRC export module implementation are:

- **Model tree preservation.** S2PLOT provides the function pushVRMLname(char *name) that can be used by the programmer to create a group in the model tree for export to VRML, our previous pathway to 3-d PDF figures. The PRC export module honours model tree names created this way and encloses subsequent geometry exported to the PRC file in a correspondingly-named group. This group name (with an automatically-generated serialized suffix that ensures uniqueness and can be obtained programmatically) can be referenced in JavaScript/s embedded in a PDF document to control the named parts of the model tree. This feature is critical: without it our implementation of volume rendering, billboards and frames in 3-d PDF would not be possible.
- **Texture compression.** Textures that are entirely opaque (i.e. textures that have no pixels with opacity less than 1.0 and are typically applied to surface meshes) are stored in the PRC file as compressed JPEG images. Textures that have transparent pixels (e.g. volume rendering textures, billboard textures) are converted to PNG images using the ImageMagick (http://www.imagemagick.org) convert tool, with their colour space limited to a maximum of 256 unique colours, using Floyd-Steinberg [14] dithering. This strategy yields savings of ∼50 per cent in file size compared to using uncompressed bitmaps of 32-bits per pixel.
- **Surface mesh compression.** Internally, S2PLOT stores surface meshes as arrays of independent facets. For export to PRC format, collections of like-coloured 3- and 4-vertex facets, each with their own vertices, vertex normals and vertex colours, are combined into single-colour meshes that comprise a list of vertices and vertex normals, that are indexed to create facets. This delivers considerable savings in storage space (vertices shared by typically three or four facets in a surface mesh are stored only once), and colours are stored once per mesh rather than per vertex or facet. Additionally, collections of facets stored as vertex-indexed meshes are loaded and rendered much faster by Adobe Reader and Adobe Acrobat, than equivalent sets of individual, unindexed facets.
- **Colour compression.** Multi-coloured surfaces, e.g. surfaces coloured by a scalar parameter mapped to a colourmap, are difficult to store efficiently and have rendered correctly and at interactive frame rates in 3-d PDF form. The S2PLOT PRC export module addresses this problem by quantizing surface colours, and creating a single mesh for each unique colour in the reduced space. In practice, this corresponds directly to applying surface mesh compression as described above, but with a relaxed criterion for determining like colours. That is, facets are combined together into a single-colour mesh when they are within a predefined distance of the current mesh colour, measured in RGB space. This procedure yields relatively compact PRC-format files, with the speed advantage of vertex-indexed meshes, with (generally) acceptable colour quantization.

### Procedure

**Figure 1:**
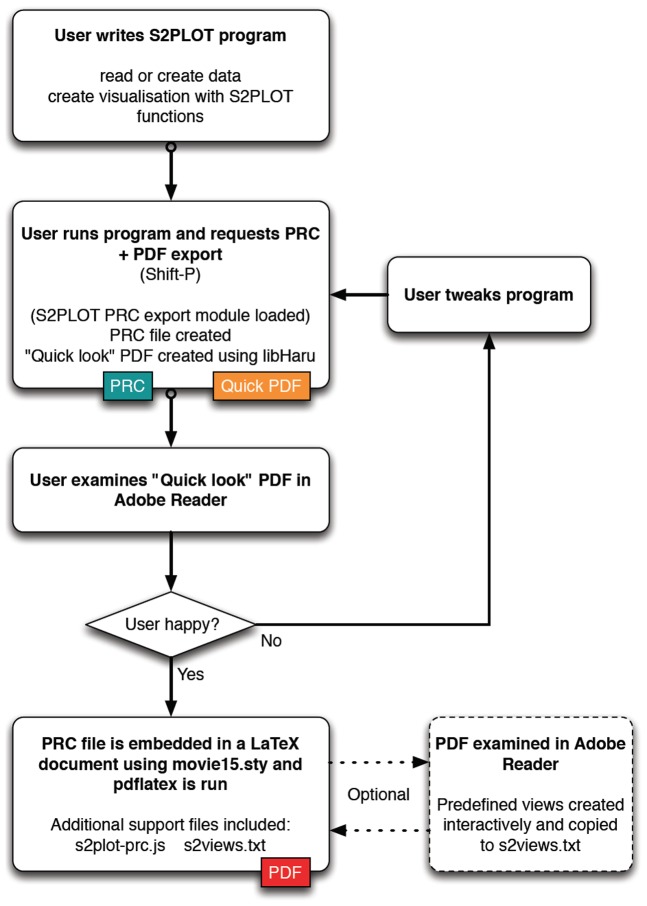
Flow diagram for creating 3-d PDF figures with S2PLOT. The final output is a PDF file generated with LaTeX, including one or more 3-d figures, embedded with movie15.sty, created via PRC export from the user's S2PLOT program.

The procedure for generating a PDF document with one or more interactive, 3-d figures is illustrated in Figure 1 and described below. Specific detail on the procedure as it pertains to the various examples in this paper will be presented alongside the examples to help the reader understand the application of the procedure to common data types such as surfaces and volumes.

#### Preparation

S2PLOT version 3.2.1 or higher (Free Software provided as source code and known to build for Linux and Mac OS X), including the S2PLOT PRC export module, is downloaded, compiled from source and installed. The user writes a new S2PLOT program (or uses an existing one) that loads or generates data, and renders it in 3-d using standard S2PLOT functions. Examples of existing S2PLOT programs available for generating 3-d PDF figures from volume and surface data are xrw2pdf and s2stl, part of the Free Software S2VOLSURF package (http://vera180.its.monash.edu.au/imgteam/index.php/projects/7-s2volsurf).

There is great flexibility at this stage of the procedure for users creating their own programs. S2PLOT is a graphics library that must be called from a driver program (usually written in the C or C++ programming language), and ostensibly provides only display-related functionality. Quite apart from calling S2PLOT functions to display data that has been read or generated by their program, the user can apply any transformation that they can express in computer program code to the data prior to mapping it to S2PLOT graphics “primitives”. For example, the user can filter (e.g. smooth) images, geometrically transform surfaces, re-project point populations and so on. And when calling S2PLOT functions, the user often has to make explicit choices about the mapping of e.g. image data values to colour, or of e.g. data parameters to physical attributes such as symbol size, arrow direction, or surface transparency.

#### Execution

The user compiles and runs their S2PLOT program, and exports a PRC file by pressing Shift-P. The S2PLOT PRC export module creates a PRC file based on the S2PLOT geometry and writes it to disk. The module also uses libHaru to create a “quicklook” PDF file with the PRC file embedded, the standard 3-d JavaScript file supplied with S2PLOT attached to the figure, and a standard set of predefined views of the model.

#### Evaluation

The user views the quicklook PDF file in Adobe Reader. If the user is happy with the output then they proceed to the “Use” step, otherwise they modify their S2PLOT program, rebuild and return to the “Execution” step.

#### Use

The user embeds the saved PRC file as a figure in an existing or new LaTeX document, using the movie15.sty or media9.sty LaTeX style, and the pdflatex program. Normally they also provide (via movie15's arguments) the s2plot-prc.js 3D JavaScript file (which provides basic keyboard controls and implements advanced techniques such as volume rendering), and a s2views.txt file which provides a standard set of views for the figure.

#### Predefined views

Further refinement can be carried out using the 3-d view reporting feature of movie15.sty to interactively create predefined views with specific parts of the geometry toggled in or out of the visualisation and/or rendered in part-specific modes, and with specific lighting choices. This is accomplished by the user inserting a movieref command (e.g. movieref[3Dgetview]{cortical_act}{Click for view output.}) in the LaTeX source file. This provides a text link, which when clicked, presents a popup window containing text defining the currently-configured view of a 3-d figure, which can be copied verbatim to the s2views.txt file. If preferred, views can be defined and added programmatically or manually to the s2views.txt file using the full node names saved by the PRC export module in the s2direct.map file.

### Validation

To validate 3-d PDF volume and surface visualisations, we generated visualisations of the same source data with corresponding, mainstream applications, under the most similar conditions we could arrange. We acquired screenshots of these “controls”, of native S2PLOT visualisations, and of 3-d PDF figures created following the procedures of this paper, with standard (operating system-included) tools, and examined the screenshots side-by-side. To assist assessment of the qualitative differences, and as a first step beyond the practice of determining that results just “look right,” we manually aligned (co-registered) comparison images in Adobe Photoshop version CS5 Extended (1990–2010, Adobe Systems Incorporated, http://www.adobe.com), and then:

- For volume rendered images, generated *difference images* by overlaying the comparison images using the *Difference* layer blending mode, converting the result to *Black & White* using an *Adjustment* layer, and smoothing with a *Box Blur* image filter of radius one pixel to suppress sub-pixel co-registration artefacts.
- For surface rendered images, applied the *Find edges* filter to the comparison images, created a mask of the edges in each image by selecting all non-white pixels, used the *Fill* function to paint the detected edge pixels a single colour, then overlaid the coloured edge images derived from the comparison images.

Comparisons made in this way are *not* quantitative, but assist in the evaluation of similarity and difference between renderings made in different packages by suppressing differences due to internal, private lighting and shading choices in the individual software packages.

## Results

### Orientation: the coordinate system, views and controls

**Figure 2:**
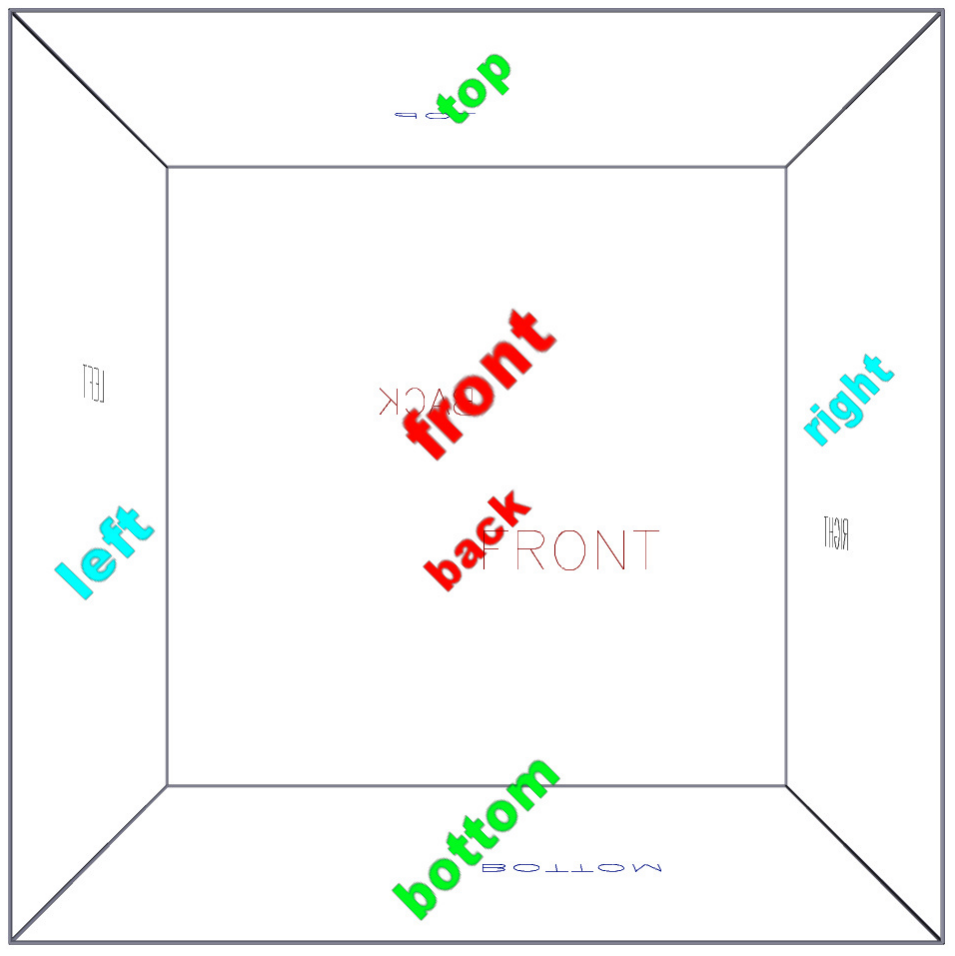
The standard S2PLOT 3-d PDF space (3-d figure). This simple figure, a wireframe cube with faces labelled, provides a simple example for becoming familiar with the standard 3-d PDF toolbar, and the default S2PLOT extensions (keyboard controls) implemented via JavaScript. When reading File S2, with a PDF reader that supports 3-d figures (i.e. Adobe Reader and Adobe Acrobat, for PC or Mac) the 3-d mode will be activated automatically. Otherwise a regular, static 2-d figure will be displayed.

To introduce the fundamental structure and user-control of the 3-d PDF figure, we present Figure 2. This figure shows a wireframe cube, with its faces marked by simple vector-based text and also by bitmap text which always faces the camera (“billboard” text). When the 3-d view is activated—which for this figure is automatic in Adobe Acrobat or Adobe Reader—the user can use the 3-d toolbar to: select preset views, select projection mode (perspective versus orthographic), and control shading and lighting properties. They can also access the model tree (the hierarchical structure of geometry primitives that constitute the 3-d scene) and enable and disable arbitrary branches and nodes. In the default mode, the user can control the camera view freely, via a primary button mouse drag for moving the camera around the model, and the mouse scrollwheel for zooming in and out. When examined in a PDF viewer that does not support 3-d figures, or when printed, a standard 2-d figure (known as a “poster image”, in this case a screenshot of the opening view of the 3-d figure) is shown.

Specific to S2PLOT-created 3-d PDF figures, are a set of keyboard controls and a set of preset views. These standard keyboard controls and preset views are listed in Table 1, and have been implemented to match the defaults for S2PLOT programs executing directly on a workstation. Accordingly, the frequent user of S2PLOT will find the same key operations and standard views in a PDF version of their 3-d scene as they will in the executable program which creates the 3-d scene.

**Table 1:** Standard S2PLOT 3-d PDF keyboard controls.

Key	View or operation
1	Front perspective
2	Back perspective
3	Left perspective
4	Right perspective
5	Top perspective
6	Bottom perspective
7	Oblique perspective
+,−	Zoom in, out
[,]	Roll camera clockwise, counter-clockwise
←, →	swing camera left, right
↑, ↓	swing camera up, down
<,>	Decrease, increase camera movement “delta”
Shift-A	Toggle autospin
/,*	Decrease, increase autospin speed

A list of the default S2PLOT extensions (keyboard controls) implemented via the provided s2plot-prc.js JavaScript.

Figure 2 also serves as an introduction to an advanced graphics primitive supported in S2PLOT 3-d PDF figures: the *billboard*. This is a 4-sided facet on which a bitmap image is displayed, however the facet is always oriented to face the camera (i.e. to be parallel to the projected image plane. We have implemented billboards in 3-d PDF by including code in the provided s2plot-prc.js JavaScript file. Leaves of the model tree that are billboards are identified at figure intialisation time, and prior to every redraw of the scene, these facets are reoriented for correct display. Billboards are ideal for displaying particles in populations (e.g. from N-body gravitational or molecular simulations) as soft, extended entities, and for displaying labels that stay upright and always face the user.

*Procedural notes:* a simple, self-contained S2PLOT program (s2views.c) was written in the C programming language. It uses standard S2PLOT functions to open the display device (s2opend), to set up the world coordinate space (s2swin) for graphics, to draw a box around the world space (s2box), and to draw simple vector-based text (ns2text). It also uses the simple S2PLOT interface (ss2ftt) to the FreeType library (http://www.freetype.org) to create neatly typeset labels and place them, rotated on the screen, always facing the camera (with function ds2vbbpr). This program example neither reads or generates data, it simply annotates the world space available for graphics display with S2PLOT. After compiling and starting the s2views program, a PRC file was exported using the Shift-P keypress. The default views and keyboard presses, implemented in the s2plot-prc.js JavaScript file and the s2views.txt text file, and default rendering and lighting modes were used for this example.

### Simple, static geometry: a 3-d figure of a protein molecule

**Figure 3:**
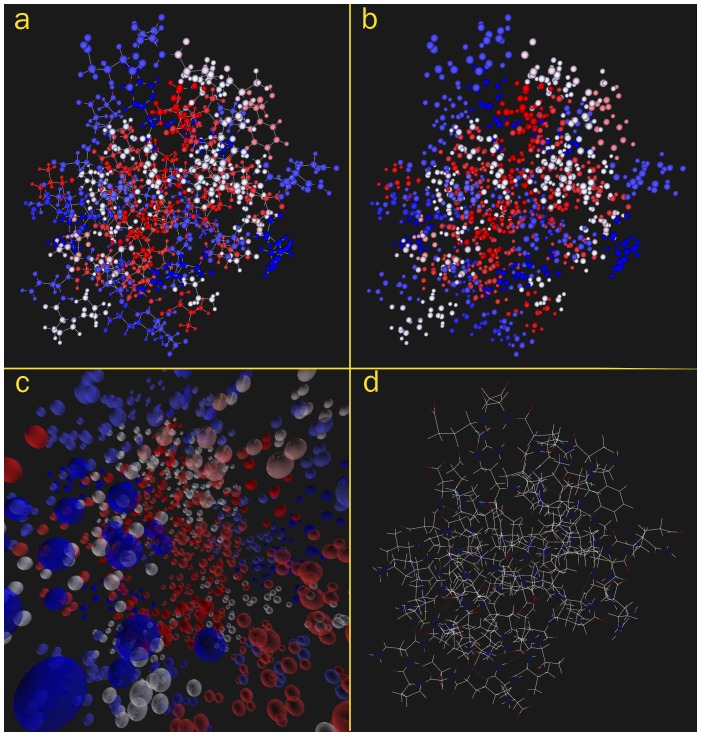
RNA polymerase alpha subunit (alpha CTD) of the *Escherichia coli* organism (3-d figure). An S2PLOT program to construct 3-d visualisations of molecules from the Protein Data Bank was used to render this part of the *E. coli* organism. Atoms are coloured according to hydropathy. When reading File S2, atoms and bonds can be toggled in and out of the visualisation by expanding the model tree, or by using hyperlinks. This figure also demonstrates the optional feature of a “poster image” which must be clicked (in File S2) to activate the 3-d figure. Data downloaded from the Protein Data Bank; original source [15].

Figure 3 presents a molecule from the Protein Data Bank (PDB) (http://www.rcsb.org/pdb), rendered by S2PLOT and converted to a 3-d PDF figure following the procedure outlined in this paper. The molecule is the RNA polymerase alpha subunit (alpha CTD) of the *Escherichia coli* organism ([15]). Bonds (atoms) can easily be toggled in and out of the display by expanding the model tree and selecting or deselecting the LINES1 (BALLS1) branches of the tree. By using the hyperlink capability of PDF, clickable links can be provided in the text to toggle particular parts of the model This figure also demonstrates the option of *requiring* the user to click on the poster image to activate the 3-d figure. In some cases this may be desirable so that readers are all presented with the 2-d poster image, regardless of the PDF reader application they are using.

*Procedural notes:* an existing S2PLOT program (smv by R. Smith, *pers. comm.*) that creates 3-d visualisations of molecule structures from the PDB, was compiled against the latest version of S2PLOT. The smv program includes code to read a PDB file, set up the S2PLOT world coordinate space, and plot the described molecular structure. It includes an in-program menu system (itself written using S2PLOT functions such as ds2ah to add interactive “handles” to the visualisation and ds2shcb to capture user input on handles) that enables the user to choose how the molecule should be coloured, whether the bonds should be displayed, and other properties. Smv was run with the selected PDB file, the visualisation was configured as required using the in-program menu, and the geometry was exported to PRC format using the standard Shift-P keypress. The standard preset views, keyboard controls, lighting and rendering scheme were ideal for this example, so no further changes were applied to the PRC or ancillary files (JavaScript, views). Two one-line JavaScript files were created to implement the clickable links in the prior paragraph.

**Figure 4:**
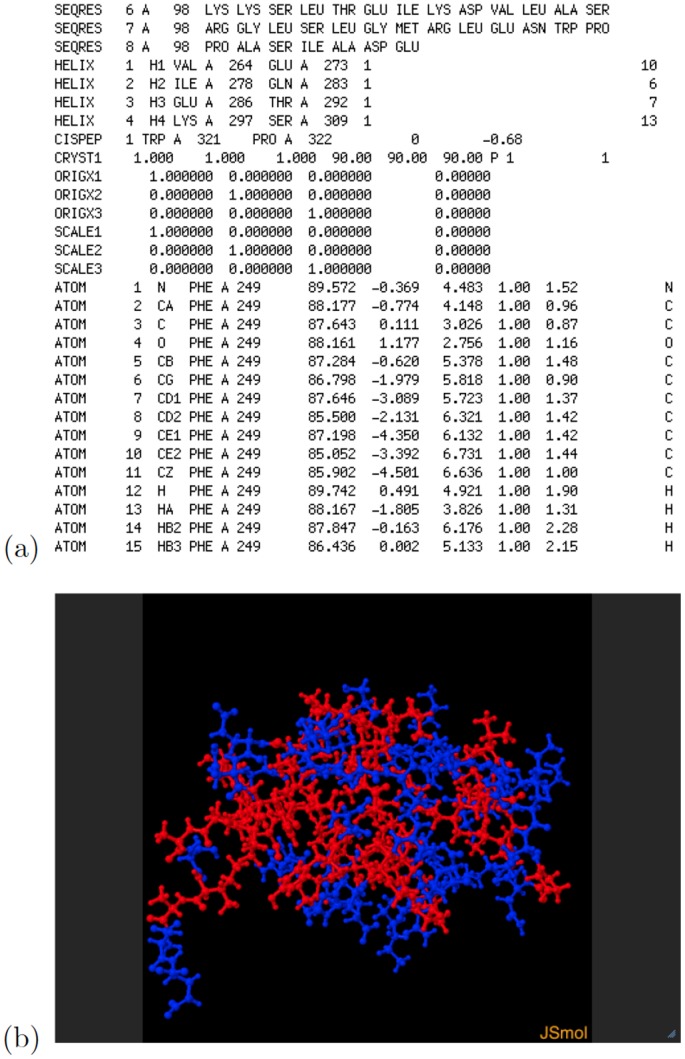
RNA polymerase alpha subunit (alpha CTD) of the *Escherichia coli* organism. (a) Excerpt of the PDB text-format description file for this protein; and (b) Jmol 3-d projection obtained at the Research Collaboratory for Structural Bioinformatics (http://home.rcsb.org) PDB site.

Figure 4 shows an excerpt of the text-format PDF file for the protein rendered in Figure 3, as well as a single 3-d projection of the same protein, generated within the PDB web pages by Jmol (an open source Java viewer for chemical structures in 3D, http://www.jmol.org) version 12.2.15.

### Structural magnetic resonance imaging

**Figure 5:**
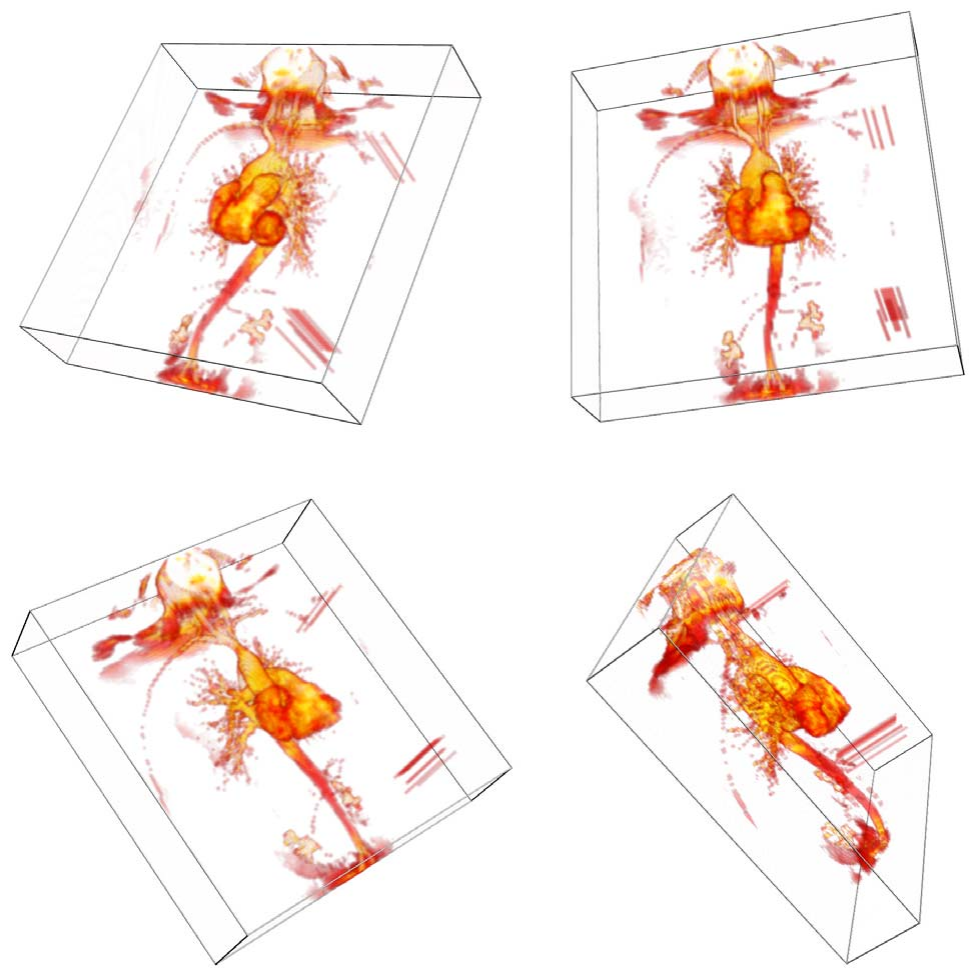
Volume rendering of a magnetic resonance angiography (MRA) image (3-d figure). The xrw2pdf utility was used to balance the opacity of each slice set to minimise difference at the (diagonal) transitions between slice sets. Data downloaded from the OsiriX Foundation and the University Hospital of Geneva; original identifier CETAUTOMATIX (Normal cardiac MRI and MRA study. Mild aortic and tricuspid valves regurgitation.) When reading File S2, click to activate the 3-d figure (when using Adobe Acrobat or Adobe Reader).

**Figure 6:**
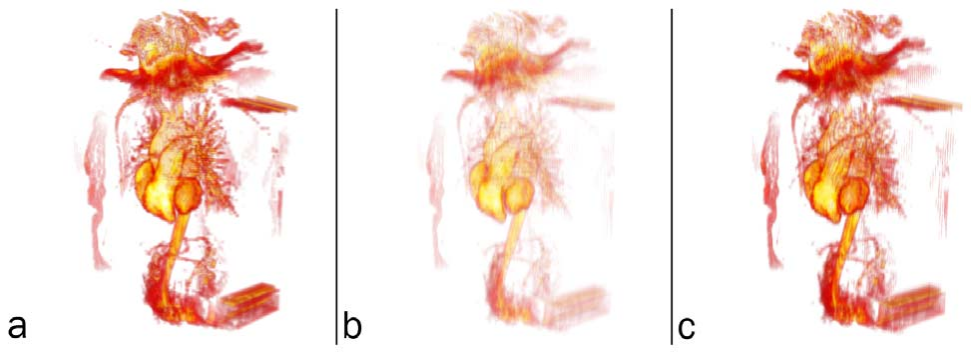
Demonstration of equalized volume rendering using opacity rescaling in xrw2pdf. (a) volume rendering of ZY slices; and volume rendering of XZ slices from a nearby viewpoint (b) without and (c) with opacity rescaling. Opacity rescaling dramatically improves the transition between renderings of different, oblique slice sets.

Figure 5 shows a volume rendering of a 3-d magnetic resonance angiograph as an interactive 3-d PDF figure. In our implementation of 3-d volume rendering in PDF, three orthogonal sets of slices through the volume are stored as pre-shaded texture images in the PDF file, but only one set is shown at any one time. JavaScript code (included in the S2PLOT distribution) is used to note camera position changes and update the selected set. This is a computationally cheap (and therefore fast) yet highly effective volume rendering technique. It does have the drawback that without manual balancing of overall transparency scaling for each slice set, the transitions between slice sets (as the camera is moved across the diagonals of the volume) can sometimes be sudden and distracting. To address this and improve upon our previous efforts (e.g. [9], [11]) we have included a command-line option in the xrw2pdf program that lets the user easily modify the opacity scaling ratios manually for the three slice sets until a satisfactory result—an *equalized* volume rendering—is obtained, prior to writing output PRC and PDF files. Figure 6 shows screen captures of the xrw2pdf program rendering nearby views but using different slice sets, without and with opacity rescaling. Saved PRC and PDF files retain the opacity rescaling.

**Figure 7:**
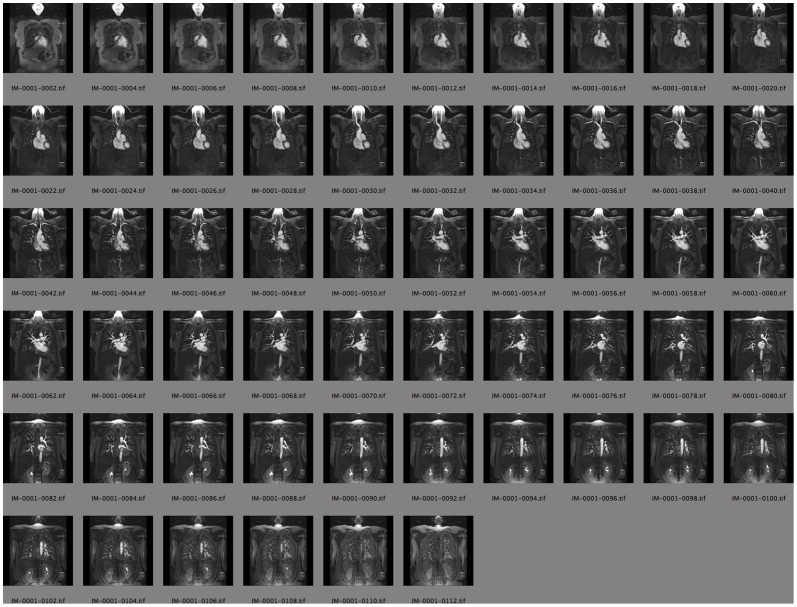
Magnetic resonance angiography slices. A subset of the 2-d image slices exported from OsiriX prior to stacking into the 3-d image used to generate Figure 5.

*Procedural notes:* we have previously described our method for volume rendering in PDF [11] and published the corresponding S2PLOT program volren. For the volume rendering in Figure 5 we adapted and expanded this program into the new S2PLOT program xrw2pdf which provides a relatively simple but capable, end-user command-line program for generating interactive 3-d volume renderings of 3-d data as PDF figures. To create this particular figure, the original data set, a multi-slice 2-d Digital Imaging and Communications in Medicine (DICOM)-format image, was opened in the OsiriX medical imaging program (version 4.1 64-bit, Pixmeo Sarl, http://www.osirix-viewer.com/). and saved as a series of TIF-format image slices. TIF slices were converted losslessly to TGA format using ImageMagick convert, and the simple tool tgastack2xrw was used to stack these slices into a single, normalised, 16-bit binary format 3-d image. (Alternatively, the DICOM image could have been converted to NIFTI image format and converted to the normalised 16-bit binary format 3-d image using nifti2xrw.) The xrw-format image was then rendered to 3-d PRC (and PDF) using xrw2pdf. Figure 7 shows a subset of the 2-d image slices exported from OsiriX prior to stacking into a 3-d image.

**Figure 8:**
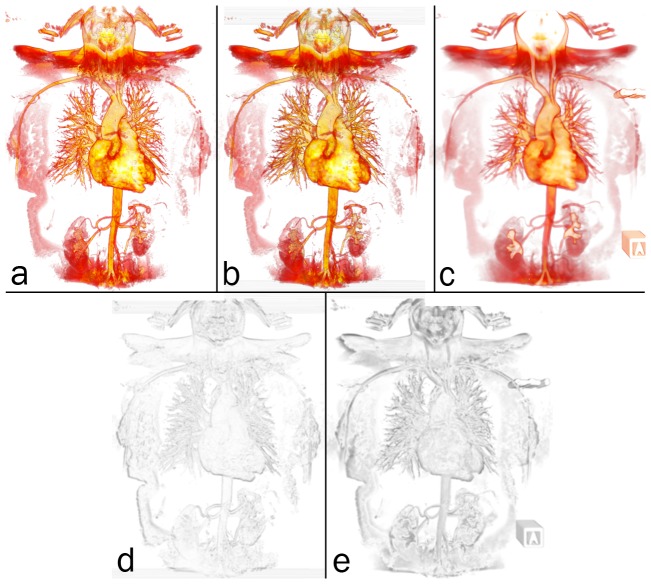
Validation of volume rendering in 3-d PDF. Top row: screen captures of the full-resolution MRA image, volume rendered in (a) S2PLOT, (b) 3-d PDF, and (c) OsiriX. Bottom row: smoothed, greyscale difference images between (d) the S2PLOT and 3-d PDF visualisations, and (e) the OsiriX and 3-d PDF visualisations. See text for further details and discussion.

An informal validation of volume rendering in PDF is given in Figure 8. The 3-d magnetic resonance angiography (MRA) image presented as the interactive, 3-d PDF Figure 5, was visualised as follows and screen captures were acquired: (1) using the S2PLOT application xrw2pdf to volume render the native resolution image (112×512×512 voxels; (2) using Adobe Reader to display the 3-d PDF figure output created by xrw2pdf from the native resolution image; and (3) using the 3-d mode of the software OsiriX. In each case, an upright, anterior coronal view was configured; and the 16-bit colour look-up table (“CLUT”) was designed in the OsiriX volume rendering display to mimic as closely as possible the “hotiron” colourmap provided by S2PLOT and mapped to the data with xrw2pdf. While subsampled data was used in the interactive 3-d Figure 5 to reduce filesize; for validation we use the full-resolution data.

### Functional magnetic resonance imaging

**Figure 9:**
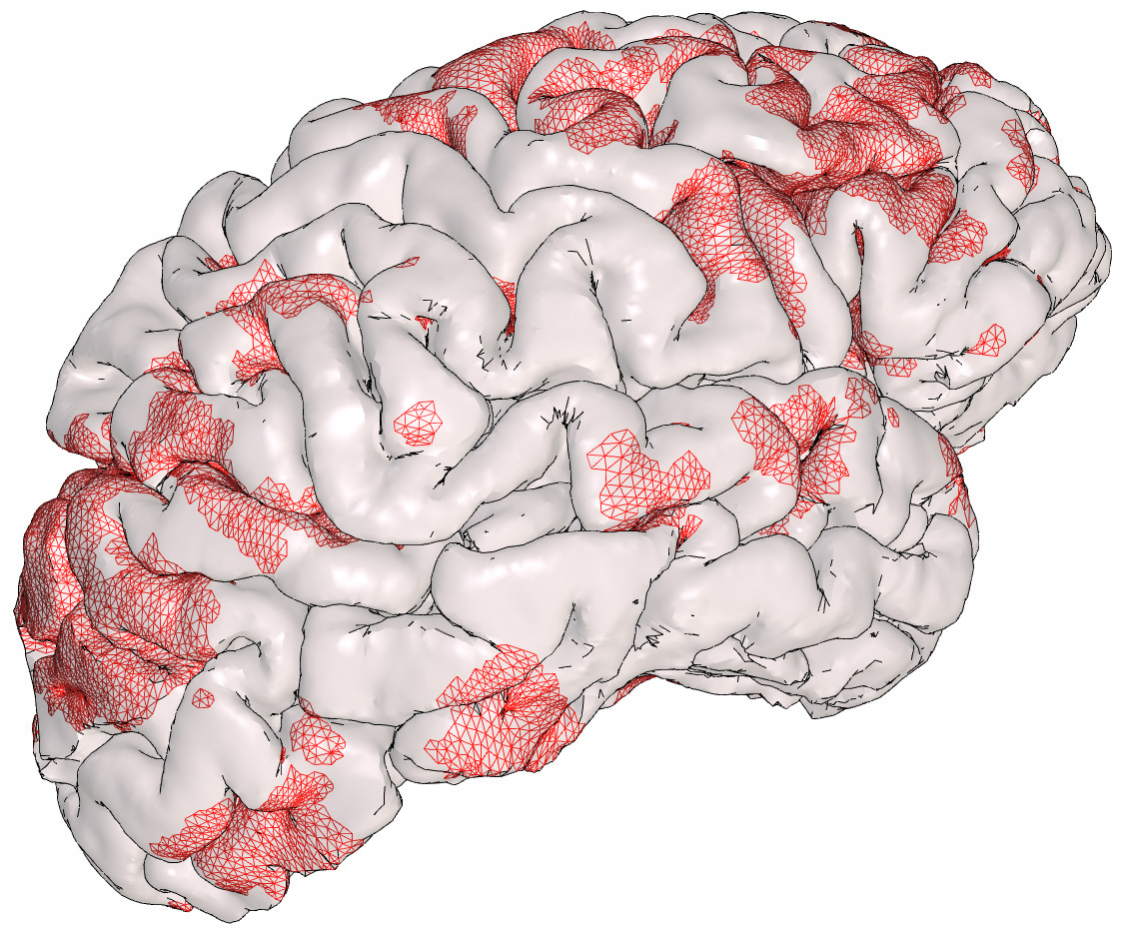
Surface rendering of the outer cerebral cortex (pial surface), and optional overlay of correlated functional activations corresponding to selected, recognised rest state networks (accessed via the “Views” menu when reading File S2). Functional MRI data set courtesy Govinda Poudel; cortical surface extraction by FreeSurfer (http://surfer.nmr.mgh.harvard.edu/).

Figure 9 shows a surface rendering of the outer cerebral cortex, or pial surface, of one hemisphere of the brain, and a small selection of rest-state networks of the brain shown as coloured sections of the surface. The cortex has a complex, folded structure that is customarily flattened for visualisation and analysis purposes [16]; using our technique we can display and share this structure in its true geometric form.

In the figure, the user can display *functionally*-correlated regions of the brain as revealed by the blood-oxygen-level-dependent (BOLD) effect [17], corresponding to known rest state networks, “painted” onto the pial surface. Selecting a specific view (e.g. [Visual network]) from the *Views* menu results in that named part of the model tree being enabled for display, and all other networks hidden from display. An additional view [Cortical surface (no activations)] is included in which all network nodes are set to be not visible.

*Procedural notes:* we were provided with a structural T1-weighted magnetic resonance (MR) image, and four thresholded functional MR images depicting independent rest-state brain networks computed by FSL Multivariate Exploratory Linear Optimized Decomposition into Independent Components (FSL MELODIC version 4.1.8, http://fsl.fmrib.ox.ac.uk/fsl/fslwiki/MELODIC), from a functional MR study (G. Poudel, *pers. comm.*). FreeSurfer (version 5.0, http://surfer.nmr.mgh.harvard.edu/) was used to compute the pial surface from the structural image, and to “paint” the rest-state network activations onto this surface. The hemisphere pial surface was exported to a FreeSurfer Triangle Surface File - ASCII Version, while the four painted regions were exported to FreeSurfer W (Weight) Files (ASCII format). An S2PLOT program was written to read the surface files (using the loadObjFromFS and loadObjWgtFromFS functions included in S2VOLSURF, and display them as coloured meshes.

The S2PLOT function pushVRMLname was used to set the name of each component for display in the model tree when the 3-d figure is displayed, and for setting up the predefined views of the different networks. Predefined views were created by editing the default s2views.txt file to include several named views, within which the pial surface and one network node are set VISIBLE = true while the other network nodes are set VISIBLE = false. To guarantee uniqueness of model tree node names in the PRC file, the PRC export code applies an auto-generated suffix to each named node. The full, suffixed node name must be used in the s2views.txt file, and so the S2PLOT PRC export code writes a utility file, s2direct.map, which includes the full node names for use in the s2views.txt.

**Figure 10:**
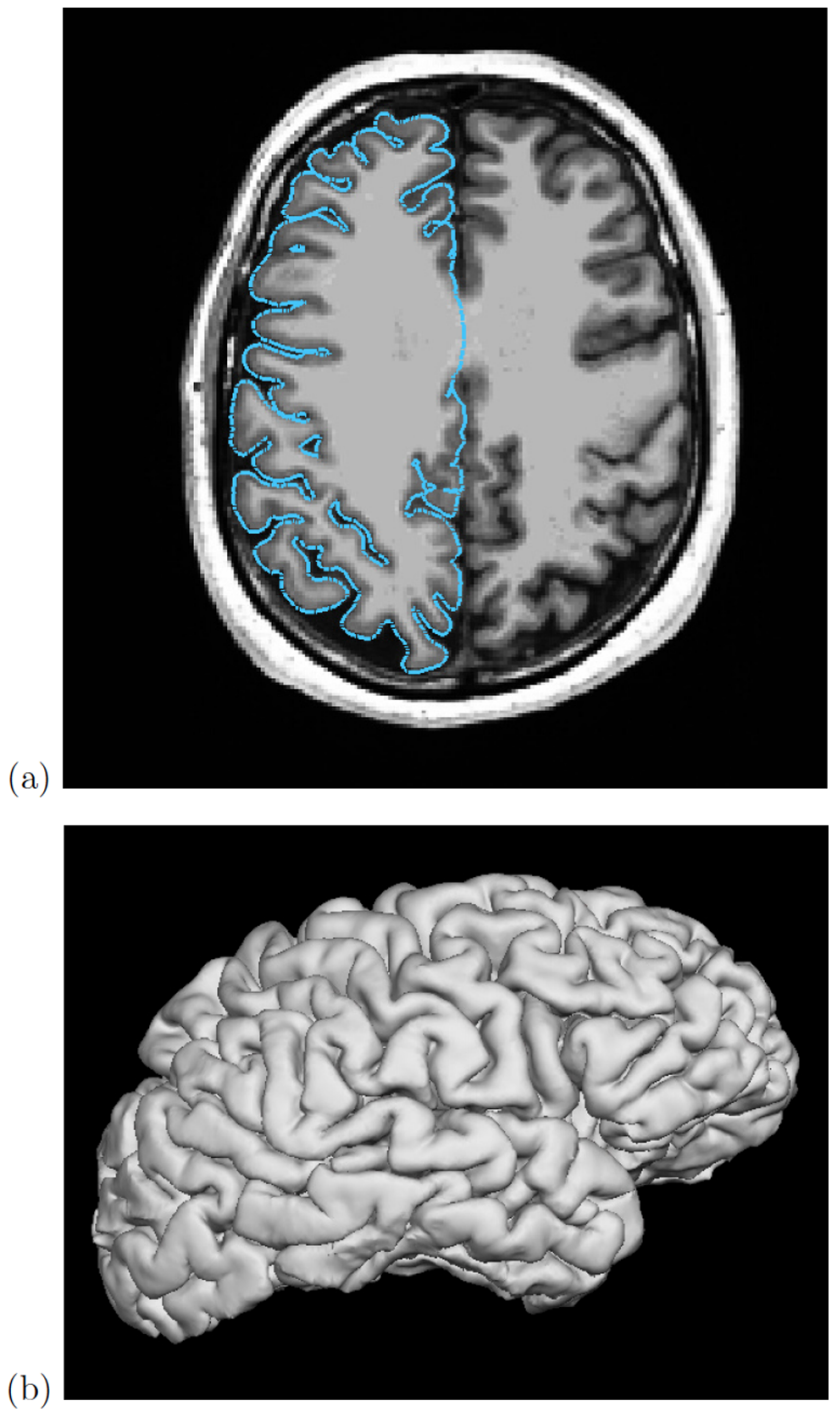
Pial surface renderings. (a) FreeSurfer tkmedit rendering of T1-weighted structural MR image and cross-section through extracted pial surface; and (b) FreeSurfer tksurfer 3-d rendering of the pial surface.

In Figure 10 the principal source data for Figure 9 is shown, as visualised by the FreeSurfer software that extracted the pial surface from the T1-weighted structural image.

### Stacked microscope sections

**Figure 11:**
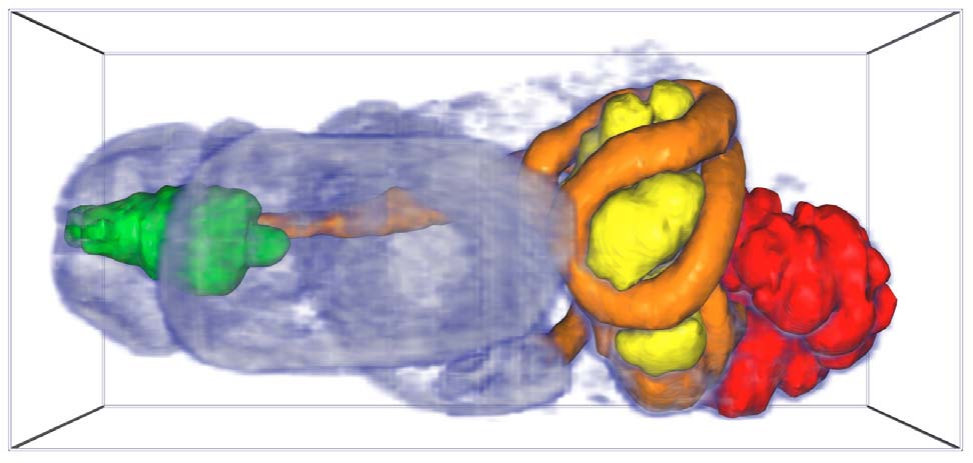
Volume rendering, and surface segmentation of the digestive tract, of a juvenile specimen of the marine pulmonate snail *Ovatella myosotis* (3-d figure). When reading File S2 the 3-d figure is automatically activated. Sample (total length: 0.86 mm) prepared as described in [23]; stacked, optical microscopy images processed and manually segmented as per [18].

Figure 11 provides an example of volume rendering, combined with manually-segmented surfaces that can be toggled in or out of the figure. The source data is a stack of 2-dimensional optical microscopy images of a series of adjacent sections of the sample—the soft parts only of a juvenile specimen of the marine pulmonate snail *Ovatella myosotis*—yielding a contiguous 3-d volume.

*Procedural notes:* (iso-)surfaces of the data were generated with the software AMIRA (version 5.4.0, Visage Imaging, Inc., San Diego, CA, USA) by manual segmentation of section data as described by [18]. The same software was used to reduce the tessellation of surface meshes to achieve a suitable file size, and to convert the surfaces to the Alias Wavefront .obj (OBJ) format.

As for Figure 5, the volume rendering component of this figure was created by assembling the slice stack into a single, normalised, 16-bit binary format 3-d image using tgastack2xrw, then displaying the volume usingthe xrw2pdf program. To overlay surfaces stored in OBJ format on the volume rendering (i.e. in the same object coordinate system space), the xrw2pdf program was enhanced to read surface(s) from one or more OBJ files and shade each surface with a nominated solid colour and transparency. A custom s2views.txt file was created (using the model tree node names in the s2direct.map file) to provide *Views* menu options to enable or disable the segmented digestive tract surfaces. As per Figure 5, the slice opacity scaling feature of the xrw2pdf utility was used to improve the smoothness of the volume rendering across the view transitions.

### Segmented anatomical structure

**Figure 12:**
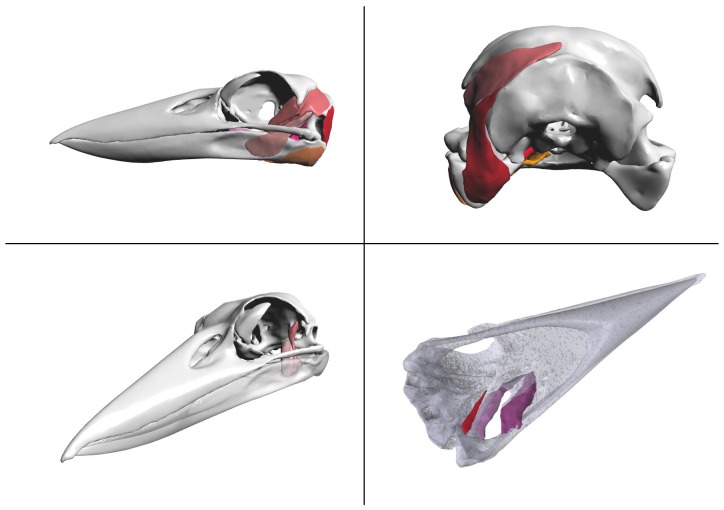
Laughing Kookaburra (*Dacelo novaeguineae*) skull and jaw anatomy (3-d figure). Manually segmented meshes simplified in MeshLab. When reading File S2, click to activate the 3-d figure (when using Adobe Acrobat or Adobe Reader).

Figure 12 presents a 3-d interactive visualisation of the jaw musculature of the Australian Laughing Kookaburra (*Dacelo novaeguineae*). We have previously constructed an anatomical model of the jaw musculature of the Kookaburra, based on a computed tomography (CT) scan of a deceased adult specimen ([19]). The Kookaburra was loaned from Australia Museum's Ornithological Collection (specimen no. BF2162), and scanned in the Newcastle Calvary Mater Hospital's Toshiba Aquilion 64 CT scanner. Segmentation of the CT images was carried out using Materialise Mimics (http://biomedical.materialise.com/mimics). The CT data was thresholded to select the areas of the image with grey scale values that represented the bone of the Kookaburra skull, omitting the soft tissue. The selected areas on each image slice were then joined using Mimics' 3D modelling tool to produce a model of the skull and jaw bones. While the soft tissue making up the kookaburra jaw muscles was visible in the CT scans, it was difficult to differentiate the separate muscles due to the low resolution of the scan. To aid in differentiating each muscle, the head of the kookaburra specimen was dissected and described. This information was then used to aid in identifying the jaw muscles in the CT scan. To create the 3D model of each of these jaw muscles Mimics' draw tool was used to manually select the edge of each muscle and join the slices to produce a 3D model. The skull and muscles were then exported in the Stereolithography (STL) file format.

*Procedural notes:* to display surfaces stored in STL format, a simple S2PLOT program, s2stl, was created to read surface(s) from one or more STL files and shade each surface with a provided solid colour and transparency. The first attempt generated a very large output PRC file (>30 MB!). The free mesh procesing software MeshLab (version 1.3.0, http://meshlab.sourceforge.net) was used to apply a quadric-based edge collapse to decrease the size of the skull mesh (the largest and most detailed component by far) sixfold to 100,000 triangles. This reduced the output PRC (and PDF) file size to under 4 MB with almost no perceptible decrease in surface detail.

Custom views were generated interactively for this figure. The PRC model was placed in a PDF file using LaTeX and the movie15.sty style file, and the movieref command was used to create a 3Dgetview clickable link. When viewing this figure in Acrobat Reader, the user can adjust all the view settings (including transparency of parts, camera view orientation, background colour and lighting) and then click on the 3Dgetview link to retrieve the exact text block to place in the s2views.txt views file to recall the configured view. This was done several times to create and store different views and configurations of transparent components in a single s2views.txt file. This file was then used in the final creation of the figure in the LaTeX document source.

**Figure 13:**
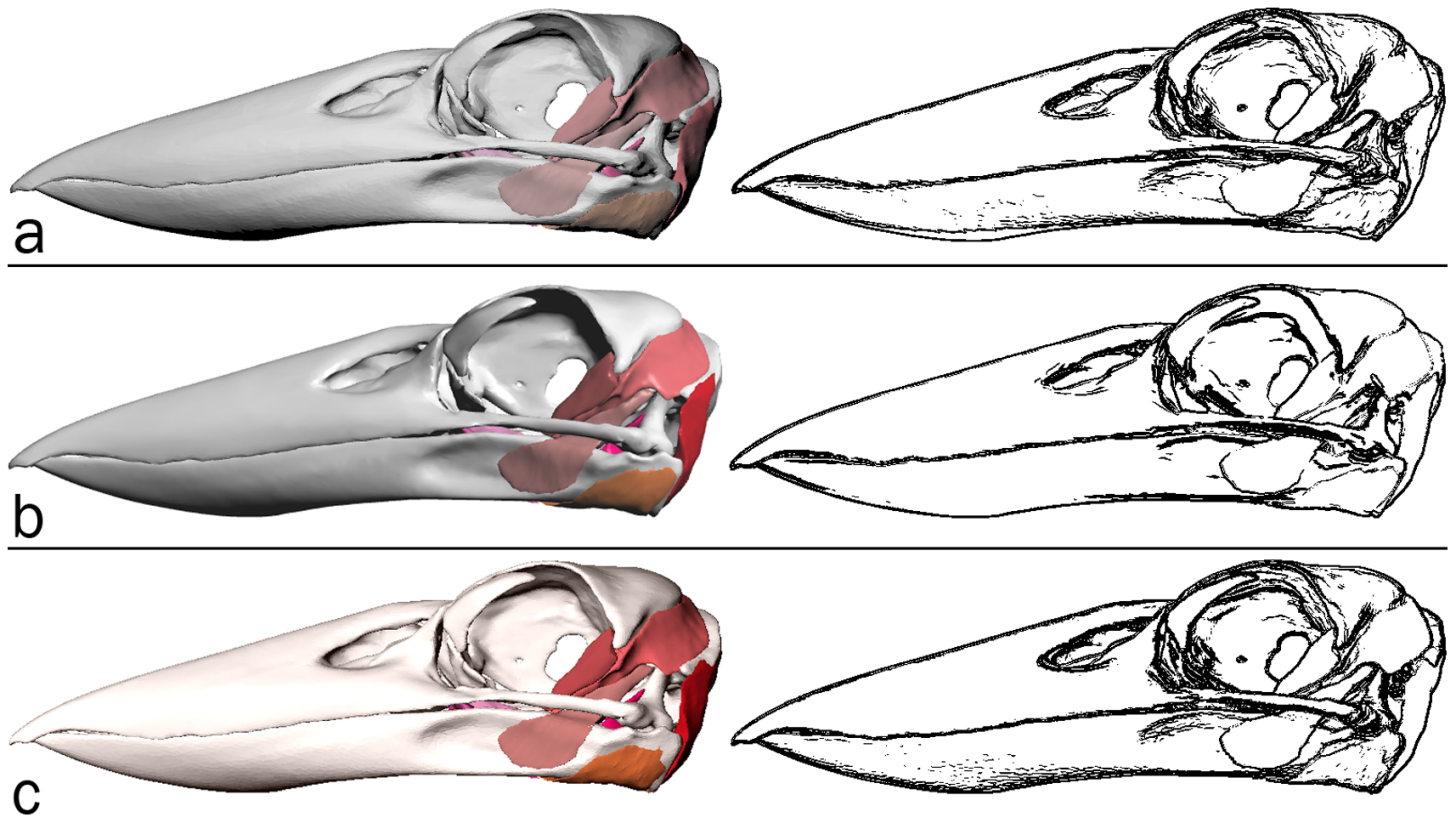
Validation of surface rendering in 3-d PDF (1). Left column: screen captures of the Laughing Kookaburra skull and jaw anatomy surface-rendered in (a) S2PLOT, (b) 3-d PDF and (c) Rhinoceros. Right column: corresponding edge-detected images. See text for further details and discussion.

**Figure 14:**
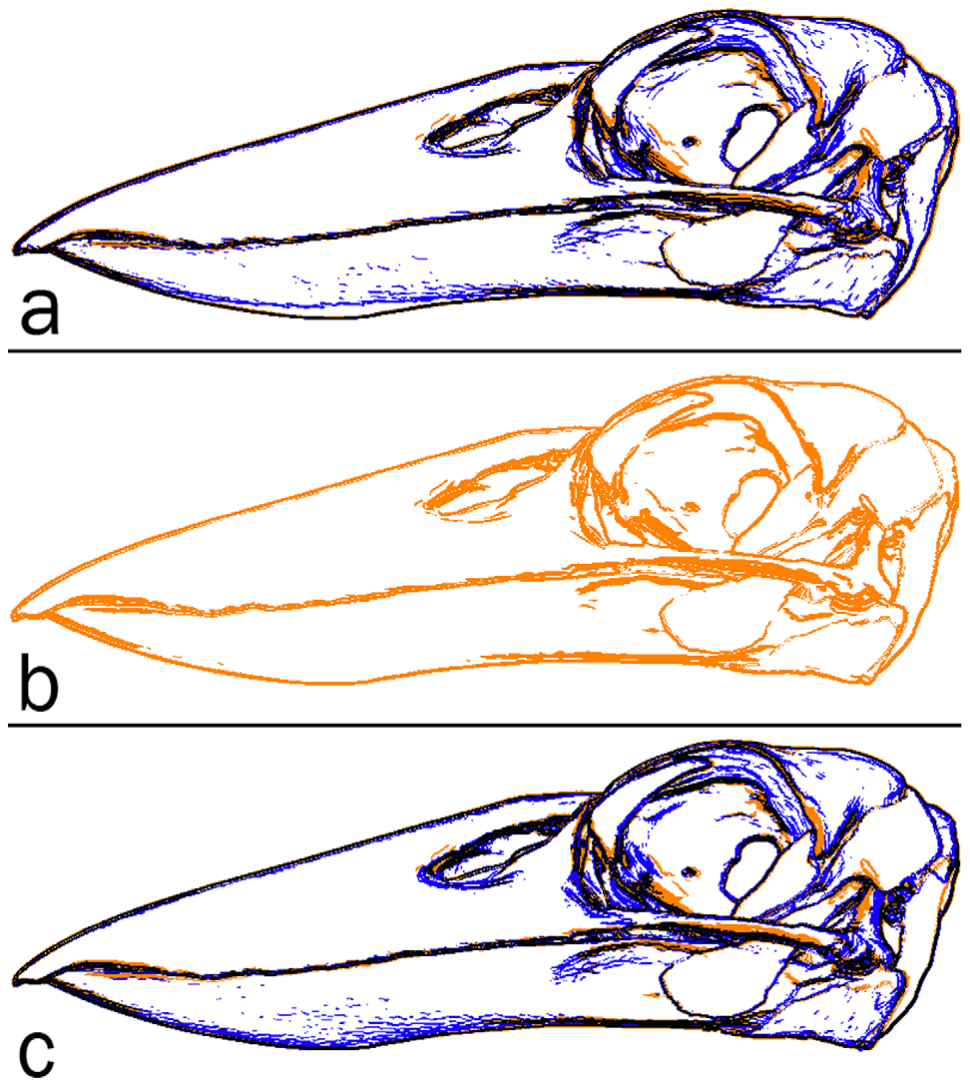
Validation of surface rendering in 3-d PDF (2). Edge-detected images from screen captures of the Laughing Kookaburra skull and jaw anatomy surface-rendered in: (a) S2PLOT (blue) and 3-d PDF (orange); (b) 3-d PDF (orange); and (c) Rhinoceros (blue) and 3-d PDF (orange). See text for further details and discussion.

An informal validation of surface rendering in PDF is given in Figures 13 and 14. The segmented surface data presented as the interactive, 3-d PDF Figure 12, was visualised as follows and screen captures were acquired: (1) using the s2stl S2PLOT program; (2) using Adobe Reader to display the 3-d PDF figure output from s2stl; and (3) using Rhinoceros (version 4.0 SR9, Robert McNeel & Associates, http://www.en.na.mcneel.com/default.htm). In each case, a left saggital view was configured; orthographic projection mode was used to eliminate perspective differences due to the confounding effects of differing virtual camera fields-of-view, distances to the subject, and magnification. It was not possible to arrange for identical lighting enviroments due to the closed nature of the Adobe Reader and Rhinoceros applications.

## Discussion

Our latest workflow for generating 3-d figures in PDF documents improves significantly upon our previous efforts [9]–[11]. Specifically we have removed the dependence on commercial software, and we have created a workflow that will suit the considerable fraction of the academic community working predominantly in the Unix environment (Mac or Linux) and using LaTeX for article preparation. Our approach is a more open and flexible framework than the (very few) commercial alternatives and supports advanced geometry types (e.g. billboards) and scientific visualisation methods (e.g. volume rendering) directly. We provide straightforward programmer-control of the hierarchical model tree, which in concert with custom JavaScript code, enables a diverse range of interactive visualisations to be imagined and constructed. To establish the technique and highlight just some of its potential, we have presented a series of figures based on research-quality data from different scientific fields. We note that by use of an appropriate LaTeX style file (e.g. prosper, http://amath.colorado.edu/documentation/LaTeX/prosper/), 3-d figures can be included in slide-based talks as well as articles, and we point out that the movie15.sty style file (and media9.sty) can also embed other media content (e.g. movies) in addition to interactive 3-d content.

### Validation

Validating new approaches for the visualisation of scientific data represents an important control [20] but is rarely acknowledged or attempted. It is made difficult by the diversity and complexity of data-to-pixel transfer functions that are applied by existing applications, and the commercially-closed nature of (some) popular visualisation software. Even for simple raster image slicing for example, without knowing and applying the *exact* mapping from data value to colour, comparison of two visualisation processes can be only qualitative. The inherently qualitative and aesthetic nature of most scientific visualisation precludes any rigourous comparison of results. Instead, “visualizations are accepted if they look more or less right”, and “the more innovative and unusual the application, the less likely … error[s] will be detected” [21].

• **Volume rendering.** In Figure 6 we demonstrate the improvement that is possible by applying opacity rescaling to 2-d slice-based volume rendering. Panel (a) shows an off-axis rendering in the S2PLOT application xrw2pdf of the ZY slice set (that is, the camera view direction is most closely aligned with the X-axis rather than the Y or Z axes. Panels (b) and (c) of Figure 6 show the resultant rendering when the camera is moved fractionally further around the object, such that the preferred slice set changes to XZ (i.e. the camera view direction now most aligned with the Y-axis), where opacity rescaling has been applied (to the XZ slice set relative to the ZY slice set) in panel (c) only. Without rescaling (b), significant global changes in the appearance of the rendering occur as the model is rotated and different slice sets are selected for rendering. These transitions are commonly distracting enough so as to disturb comfort in viewing the model and comprehension thereof. Rescaling, available on the xrw2pdf command-line, can improve the rendering to the point where many observers will no longer notice the transitions between slice sets, as comparing (a) and (c) demonstrates. It is important to note that opacity rescaling is *not* linear (overall opacity accumulates exponentially along sight-lines for standard OpenGL blending modes used in slice-based volume rendering) and so the preferred rescaling cannot be calculated. Instead it is found by manual user setting of the relative scaling between the three independent slice sets, with a good starting point for the rescaling values being the ratios of the relative axis lengths (pixel dimensions) of the data volume being rendered. Volume rendering using 3-d textures, which avoids all need for opacity rescaling by virtue of OpenGL's texture resampling capabilities, is available in xrw2pdf but cannot be implemented in 3-d PDF figures due to lack of support for 3-d textures in the 3-d PDF extension.

In Figure 8, we show that the overall differences between volume renderings of the same data in panels (a) S2PLOT, (b) 3-d PDF, and (c) OsiriX, are small. The formal difference between the S2PLOT and 3-d PDF images—panel (d)—is insignificant. This is expected, since the same volume rendering operation (OpenGL-based texture sampling and blending) is being applied in both renderings, to *identical* texture data. The OsiriX and 3-d PDF images show more difference—panel (e)—mostly associated with the edges of prominent image features. Texture sampling and blending are different in this case (OsiriX uses 3-d texture-based rendering, 3-d PDF uses 2-d texture-based rendering; although for this face-on view the difference would be subtle), and while the transfer function from data value to voxel colour and opacity has been constructed in the OsiriX 16-bit editor to be similar to that used for the 3-d PDF figure, it is *not* identical. These known differences in the rendering satisfactorily explain the differences observed. Qualitatively, the OsiriX rendering is smoother, but arguably less detailed than the S2PLOT and 3-d PDF renderings. The above notwithstanding, the principal outcome is that the 3-d PDF volume rendering offers as faithful and accurate representation of the volume data as other packages might, in the context of variable rendering techniques, data transfer functions, and data pre-processing methods.

• **Surface rendering.** In Figure 13 we present the surface-based anatomical model of the jaw musculature of the Australian Laughing Kookaburra as rendered in panel (a) by S2PLOT, (b) 3-d PDF, and (c) Rhinoceros. Of note are the apparent differences in default lighting conditions, and surface “materials”. For example, the Rhinoceros rendering suggests more reflective surfaces and/or brighter directional lighting, than present in the S2PLOT or 3-d PDF scene; while colours are significantly less saturated in the S2PLOT rendering. Figure 14 attempts to display the *geometric* differences between the renderings of the Kookaburra jaw anatomy, with effects due to the lighting and material environment suppressed. Clearly, all three models show good agreement in terms of feature edges, but minor differences remain, predominantly falling in deep shadow areas and in highly-curved parts of the surfaces. Computed differences in images of scientific data surface rendered in different packages clearly have limited value, and this observation is linked to the scant presence in the academic literature of quantitative validation and comparison studies of surface visualisation techniques. We acknowledge that different software will always prefer different material and lighting conventions, and simply offer the commentary that in comparison to Rhinoceros and S2PLOT, there are no identifable flaws with the 3-d PDF surface rendering that would preclude its use in practice.

Unfortunately, the rendering of transparent surfaces in 3-d PDF is not done well. We believe that the viewing applications (Adobe Reader and Adobe Acrobat) are not sorting the individual facets that make up transparent meshes, and accordingly, back-to-front drawing order, which is necessary for correct drawing of transparent surfaces, is not determined. The effect of this can be observed in Figure 12 by selecting the view *Dorsal Pterygoid muscles* or *Ventral Pterygoid muscles* and rotating the model. The mottled presentation of the cranium and mandible bone surfaces results from the triangular facets that make up the surface being drawn in an effectively random order: in any view, some of the facets on the distant surface are (correctly) drawn before the overlaying near surface facets, while other facets are (incorrectly) drawn *after* the near surface facets that overlay them. Not only are single surfaces drawn incorrectly, but folded and/or multiple transparent meshes that interleave along the viewing direction will generally not be rendered properly. A work-around exists: in place of storing multi-faceted meshes in the PRC file, it is possible to store a large list of unstructured transparent facets. These will be rendered correctly, but the cost in terms of file size, rendering frame rate, and especially 3-d figure “initialisation” time can be prohibitive, even for a relatively small set of facets (e.g. a few thousand). The long-term solution is for the viewing application to globally sort all transparent facets in all meshes, before rendering the current camera view.

### Alternatives

The published use of 3-d PDF outside of the computer-aided design/computer-aided manufacturing (CAD/CAM) industry, is extremely limited. Hence an in-depth comparison of the 3-d PDF output of S2PLOT with the output of other programs is neither feasible nor useful. For academic scientific use, where open knowledge and accuracy in transferring raw image data to visualisations is more important than (i) aesthetics and (ii) the protection of commercial intellectual property, the selection of a tool to create a 3-d PDF figure is defined at present by the data type.

For users wishing to create 3-d PDF figures of analytic surfaces, or expertly-annotated, “traditional” 3-d plots (information visualisations), **Asymptote** [22] is the appropriate tool. Asymptote can create PRC-format files for use with LaTeX and movie15.sty (or media9.sty) and can also directly create PDF files with embedded 3-d figures. Asymptote is a program whose base data elements are bezier surfaces and patches. The opposite approach is taken by MathGL (http://mathgl.sourceforge.net), a library that outputs tessellated data (line segments and triangles). But it is primarily intended to make bitmap visualizations of massive data sets, while 3D PDF output was introduced as an afterthought and its quality has been often sacrificed to simplify and speed up its built-in renderer.

For users working exclusively with meshes, **MeshLab** can export Universal 3D (U3D) format, which can also be embedded in PDF documents using LaTeX. S2PLOT's strengths are support for 3-d imaging data and large point data sets, direct scientific visualisation (e.g. volume rendering), and the ability to customise and generate almost any visualisation in a 3-d PDF figure by using both custom S2PLOT code and custom 3-d JavaScript code. *The ability to structure and name the model tree in S2PLOT, and therefore control the visualisation to be displayed in Adobe Reader via custom 3-d JavaScript code, is currently unique.*

### Poster images

It is worth reminding the reader that 3-d PDF figures do not preclude the use of normal 2-d figures in a document. Indeed, the poster image can and should be used to provide one or more traditional 2-d images for PDF reader applications that do not support 3-d figures, or for printing. Including 3-d figures in a PDF file *adds* capability for publication and communication and need not impact or change any existing practices oriented towards static media (paper). We have used poster images in all the figures of this paper.

### File size

The inclusion of 3-d figures in a PDF document increases the file size. However, judicious use of resampling (for 3-d volumetric images) and mesh simplification (for surfaces) can usually deliver substantial file size savings without compromising the quality of the rendering or impacting on comprehension of the data. In any case, we advocate that larger files are entirely warranted when the use of 3-d figures systematically improves the communication and comprehension of multi-dimensional datasets, models and research results.

### Publishing best practice inertia

We have been developing 3-d PDF figure publishing techniques for nearly five years. We have experienced scientific publishers who are extremely keen to explore how this technology can provide new features for their user communities, and we have experienced the opposite. The most common issue is that the Adobe PDF specification which includes the 3-d PDF “annotation” feature (PDF/E, which we use to deliver 3-d figures in PDF) is not considered by publishers and librarians to be “archival” (PDF/A). We hope that increased uptake and use of 3-d figures in PDF by the science community will motivate publishers to work towards an archival PDF specification that includes 3-d figures, or at least develop publishing practices that support the use of PDF/E documents alongside PDF/A.

### Viewers

Presently, the only applications that support the display of 3-d figures in PDF documents are Adobe Acrobat and Adobe Reader on desktop platforms (Apple Macintosh OS X, Microsoft Windows, Linux). Adobe Reader does not support 3-d PDF figures on the popular iOS (iPhone, iPod Touch, iPad) platform; we are not aware of any other useable 3-d PDF software for this platform, although a recently-released product, “3D PDF Reader” for iPhone, iPod Touch and iPad (https://itunes.apple.com/us/app/3d-pdf-reader/id569307672) created by Tech Soft 3D, is able to display PRC- and U3D-format 3-d models embedded in PDF documents. However, 3D PDF Reader does not support JavaScript 3D, nor does it support documents with multiple 3-d figures. Accordingly it is (presently) not useful for anything but the simplest S2PLOT-generated 3-d PDF figures.. While it would be possible to write an (incomplete) PRC *reader* and display the contained 3-d geometry for almost any platform supporting OpenGL (as Tech Soft 3D have done for the iOS platform), the overwhelmingly difficult task would to implement the full 3-d JavaScript API so that custom visualisations such as those produced by S2PLOT could be rendered correctly.

### Concluding remarks

At present 3-d PDF figures in contemporary scientific literature are an unusual, novel occurrence. Our work aims to lower the barriers to using this technology, and we hope that the new workflow presented in this paper will encourage the community to begin using this technology in earnest, to advance scientific communication. The latest S2PLOT software which includes the PRC export module, can be obtained from http://code.google.com/p/s2plot/. The S2VOLSURF kit which includes the xrw2pdf and s2stl S2PLOT programs mentioned in this paper is freely available from http://code.google.com/p/s2volsurf/.

## Supporting Information

File S1 **A brief history of 3-d PDF.**
(PDF)

File S2 **Supplementary download paper with embedded 3-d figures.**
(PDF)
