# Supplementary material for: Embedding and Publishing Interactive, 3-Dimensional, Scientific Figures in Portable Document Format (PDF) Files
Source: PLoS One. 2013 Sep 25;8(9):e69446. doi: 10.1371/journal.pone.0069446 (PMC3783416; doi:10.1371/journal.pone.0069446)
Supplement: File S1 — A brief history of 3-d PDF. (PDF) [file pone.0069446.s001.pdf]

Embedding and publishing interactive, 3-dimensional, scientific figures in Portable Document Format (PDF) files

David G. Barnes[1*,2,3], Michail Vidiassov[4], Bernhard Ruthensteiner[5], Christopher J. Fluke[6], Michelle R. Quayle[7], Colin R. McHenry[7]
1 Monash Biomedical Imaging, Monash University, Clayton, Victoria, Australia
2 Monash e-Research Centre, Monash University, Clayton, Victoria, Australia
3 Faculty of Information Technology, Monash University, Clayton, Victoria, Australia
4 Institute of Asian and African Studies, Moscow State University, Moscow, Russia
5 Section Evertebrata varia, Zoologische Staatssammlung München, München, Bavaria, Germany
6 Centre for Astrophysics & Supercomputing, Swinburne University of Technology, Hawthorn, Victoria, Australia
7 Department of Anatomy and Developmental Biology, School of Biomedical Sciences, Monash University, Clayton, Victoria, Australia
∗ E-mail: david.g.barnes@monash.edu

Appendix 1. A brief history of 3-d PDF

The history of 3D PDF starts in 2004, when Intel led an ambitious effort to promote its U3D (Universal 3D) format as a "JPEG for 3D", "an open and extensible format for the sharing and visualization of 3D data in any mainstream application", in order "to support the re-purposing of existing 3D CAD data for use in other applications" (Interview with Richard D. Benoit, `http://www.3d-test.com/interviews/intel_3.htm`). The format itself was based on technology developed by Intel since the late 1990s (see `http://www.okino.com/conv/imp_u3d.htm` for a detailed history). To back the idea, Intel pushed U3D through ECMA standardization, updated and made available a C++ reference SDK, and assembled the 3D Industry Forum (3DIF) of interested companies.

Adobe was among the 3DIF members. It entered the engineering V&M (view and mark-up) market with its PDF-based tools, but lack of native 3-d was a significant disadvantage in a field with such established players as Autodesk with its DWF (Design Web Format). The PDF format specification version 1.6 (2004) included 3-d "annotations," a mechanism to embed 3-d models in U3D format into PDF files, and Adobe Reader 7 and Acrobat 7 Professional implemented it (early 2005). But real change was brought by Acrobat 3D (early 2006), which enhanced Acrobat 7 Professional with converters from many 3-d (including major CAD) formats to U3D and a graphical 3-d editor. Adobe's implementation of a U3D viewer was not based on the Intel code, but on the Deep Exploration product by Right Hemisphere: the implementation of the U3D standard was not complete and proprietary extensions were used.

Unfortunately, the development of U3D ceased in 2006: the forum was disbanded, essential agreed-upon changes to U3D format (like non-uniform rational b-splines) were not implemented, plans to make U3D an ISO standard were dropped, and the U3D SDK was frozen. Intel lost interest in the project and Adobe did not pick up the slack. No other vendor but Adobe and Right Hemisphere implemented viewing or embedding of U3D in its data, but some products can export U3D or PDF with embedded U3D for viewing in Adobe programs.

In 2006 Adobe bought TTF, a French provider of CAD file conversion tools, that had its own rich internal format called PRC (Product Representation Compact). The ability to embed PRC models first appeared in Acrobat 3D version 8 (end 2006), but TTF technology was fully integrated only into Acrobat 9 Pro Extended (mid 2008), including not only a new set of CAD file importers and a new 3-d editor/reviewer (3D Reviewer from TTF replaced 3D Toolkit from Right Hemisphere), but was also accompanied with an SDK that allowed the programmatic creation of 3-d annotations and 3-d models (although 3-d libraries were only for Windows and not open). On the other hand, the 3-d viewer built into Acrobat and Reader did not change much, although it got partial support for displaying PRC models.

The PDF format specification version 1.7 (2004), that became ISO standard 32000 in 2008, does not include PRC 3-d annotations. Adobe documented them in Adobe Supplement to the ISO 32000 (2008), published the PRC format specification as part of the Acrobat SDK, and initiated the process of making PRC an ISO standard.

In another change of strategy at the end of 2009 Adobe drastically scaled back its effort in the engineering publishing market, laid off staff from 3D team and in May 2010 transferred the code, its TTF team and all PRC development to Tech Soft 3D ("The future of 3D PDF", Martyn Day, AEC Magazine, 25 July 2011, `http://aecmag.com/index.php?option=content&task=view&id=444`) while stressing that "Adobe is fully committed to supporting 3D viewing and interaction capabilities within Adobe Reader and Acrobat" (`http://blogs.adobe.com/conversations/2010/05/3d_solutions_update.html`). So now no version of Adobe Acrobat X (end 2010) and XI (end 2012) comes with 3-d format translation tools, SDK or 3-d editor, but those missing parts, somewhat updated since Acrobat 9, can be purchased from Tetra 4D as "3D PDF Converter". While Tetra 4D is the front company for selling Tech Soft 3D (former Adobe, former TTF) 3D PDF creation technology to end users, the front organization behind 3D PDF and PRC as open formats is the 3D PDF Consortium.

The Universal 3D (U3D) format. The main types of data that can be stored in U3D file and viewed in Adobe products are triangle meshes, lines and points (parts of the standard considered irrelevant to engineering, like skins and bones, were not implemented in Adobe viewer, the less relevant parts, like lines and points, are partially supported). Since the U3D format has been in use by Adobe for a relatively long time and the Intel sample SDK is free and open (and includes a converter to U3D from a simple VRML-like text format IDTF), several third-party developers have supported U3D export in their programs. The most important commercial U3D tool is SAP Visual Enterprise Author (formerly Right Hemisphere Deep Exploration) which can export *and* import U3D into an editor, making it an extremely useful developer tool.

Another very useful tool is the free DAZ Studio (`http://www.daz3d.com/products/daz-studio/daz-studio-what-is-daz-studio/`), a 3-d posing application. Among its many features (which are mainly oriented to handling 3-d cartoon characters) it can import models in the widely-used Wavefront OBJ format and export to U3D. The best known open source tool with U3D export capability is the MeshLab mesh processing tool. The U3D format is codified as an ECMA-363 standard, but its implementation by Adobe and Right Hemisphere is significantly limited.

The Product Representation Compact (PRC) format. Since the PRC format was developed as an internal format for a CAD translation tool, it is able to store many types of elements in an elaborate scene graph. Basic types include points, line and curve sets, meshes of triangles and NURBS patches, that (optionally) may be subject to efficient lossy compression. Complex types range from spheres and cones to tubes, surfaces of revolution and function plots. The PRC library originating from TTF is naturally the reference implementation for reading and writing PRC format files (the file format was developed for the library, not vice versa). But it is commercial, closed-source and (for end-users) Windows-only, while the PRC format is so rich and complex, that no other complete implementation is likely to arise. The Adobe 3D viewer (in the Adobe Reader and Acrobat applications) only implements an (unspecified) subset of the PRC specification.

The 3-d enabled PDF in general. Although described in open specifications, the 3-d features of PDF in fact remain proprietary, since the Adobe 3-d viewer built into Acrobat and Reader is the only viewer available. Although incomplete, imperfectly documented and not always consistent in rendering, that viewer provides relatively stable results for core functionality, required for publishing CAD data. In view of that features and limitations we consider 3-d in PDF a valuable tool to provide supplementary 3-d representation of scientfic data for the considerable time period while PDF remains one of the major formats of electronic scientific publishing. In the long run strong competition to 3-d PDF from 3-d enabled formats that were designed with on-screen and on-line viewing from the ground up is expected. Even now, while 3-d PDF support on iOS is as described above, preliminary and patchy, Apple iBooks already

has native support for 3-d models (COLLADA files embedded in a proprietary EPUB-based format). An open-standard and open-source contender may be HTML5 with embedded X3D, powered by WebGL. Although there is a lot of activity in the WebGL field at present, experience with VRML shows that 3-d web has the ability to stay "just around the corner" for years. Accordingly, 3-d PDF will likely remain a reasonable solution for the medium- to long-term.
